# Prolonged Turnaround Time of the Hematology Laboratory and Related Factors in the South Gondar Zone, North West Ethiopia, Multi‐Centered Study

**DOI:** 10.1002/jcla.70198

**Published:** 2026-03-15

**Authors:** Birhanemaskal Malkamu, Getaneh Atikilt Yemata, Andargachew Almaw, Ayenew Berhan, Ayenew Assefa, Alemie Fentie, Birhanu Gete, Biruk Legese, Fanta Obsa, Gebyaw Arega, Habtamu Demelash, Tahir Eyayu, Teklehaimanot Kiros, Mahider Shimelis Feyisa, Mulat Erkihun, Mitikie Wondmagegn, Yenealem Solomon, Shewaneh Damtie, Tegenaw Tiruneh, Girum Tesfaye

**Affiliations:** ^1^ Department of Medical Laboratory Sciences College of Health Sciences, Debre Tabor University Debre Tabor Ethiopia; ^2^ Department of Public Health College of Health Sciences, Debre Tabor University Debre Tabor Ethiopia; ^3^ School of Medical Laboratory Sciences, Faculty of Health Sciences Jimma University Jimma Ethiopia

**Keywords:** Ethiopia, hematology, medical laboratory, prolonged, turnaround time

## Abstract

**Background:**

Turnaround time refers to the time it takes from receiving a sample in the lab to delivering test results to clinicians or stakeholders. This study aimed to determine the magnitude of prolonged turnaround time in the hematology laboratory of three selected hospitals in the South Gondar zone, North West Ethiopia.

**Methodology:**

An institutional‐based cross‐sectional study was conducted from September to December 2023 in the hematology laboratory of selected South Gondar zone hospitals. Debre Tabor's Comprehensive Specialized Hospital was selected purposefully as the sole referral hospital, while Addis Zemen and Nefas Mewcha primary hospitals were randomly selected. The final sample size was 2401. Missing values (*n* = 70) were managed using the deletion method, assuming data were Missing completely at Random (MCAR). The final dataset included 2331 dataset.

**Result:**

Of the 2331 samples, 807 (34.6%: 95% CI 32.8%–36.6%) had prolonged turnaround time. Poor sample condition caused delays in 382 (16.4%) samples. Workday of the weeks, clotted blood samples, and blood fill levels were significantly associated with prolonged TAT.

**Conclusion:**

The magnitude of prolonged TAT in hematology laboratories is high. The delays are primarily driven by preanalytical factors, specifically poor sample condition (clotting and improper fill levels) and timing of collection (Fridays). Interventions should focus on enhancing preanalytical sample quality management and optimizing laboratory workflows to reduce prolonged TAT.

## Graphical abstract

Long turnaround times were prevalent in the hematology laboratories of South Gonder zone hospitals, predominantly as a result of staff incompetence. Clotted blood samples, low blood fill levels, and the day of the week were significant factors linked to delays, with samples collected on Friday having a higher likelihood of prolonged turnaround time (TAT), while those from Tuesday to Thursday had lower odds of a delay compared to Monday. Improving employee performance through ongoing training and frequent performance reviews is crucial to resolving this issue.

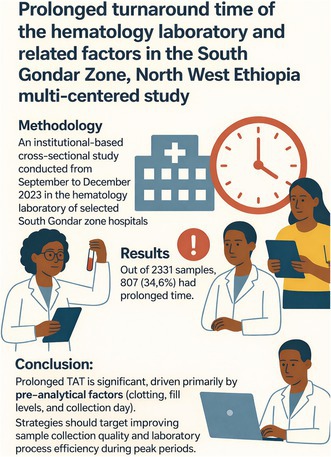

**Abbreviations**
AOR: Adjusted Odds Ratio
CBC: Complete Blood Count
CI: Confidence Interval
COR: Crude Odds Ratio
DTCSH: Debre Tabor Comprehensive Specialized Hospital
EDTA: Ethylene Diamine Tetra Acetic Acid
ESR: Erythrocyte Sedimentation Rate
IRB: Institutional Review Board
STAT: Surgical Turnaround Time
TAT: Turnaround Time

## 1 Introduction

Historically, laboratories have limited quality services to technical or analytical quality, focusing on imprecision and inaccuracy targets [1]. On the other hand, clinicians are concerned with service quality, which includes overall test error (precision and accuracy), as well as accessibility, affordability, relevance, and timeliness of the service. Clinicians like to have a service that is swift, dependable, and affordable [2]. Timeliness is likely the most significant of these criteria to the physician, who may be willing to forgo analytical quality for a shorter turnaround time (TAT). The turnaround time (TAT), which is used in hospitals to monitor laboratory performance, is defined as the amount of time it takes from order receipt to result reporting [2, 3].

One of the metrics used to evaluate a laboratory's performance is TAT. The laboratory turnaround time is the time it takes from when a test is ordered until the results are reported. The accuracy and precision of the test results are typically prioritized by laboratories as quality service goals [4]. Turnaround time reduction is critical for early diagnosis and treatment, which reduces patients' hospital stays and, as a result, improves their satisfaction and safety [5]. The turnaround time is a helpful indicator of laboratory efficiency and a measure of service outcomes, particularly for stat testing. Despite the ongoing drop in TAT, clinicians continue to demand speedier service [2, 6].

The type of test performed, the priority of the examination, the type of patient for whom the test is intended, and the activities all have an impact on TAT [7]. Clinicians and laboratory experts have varying meanings for the term “TAT” when referring to a specific test. Turnaround time refers to the time it takes from receiving a sample in the lab to creating a report for laboratory experts. Clinicians, on the other hand, measure TAT from the moment the test is requested to the time the report is received [4, 8]. Turnaround time definitions differ even within laboratories in terms of start and finish positions. For the purpose of this study, laboratory Turnaround Time (TAT) is explicitly defined as the time interval from the documented receipt of a patient sample within the specimen reception area of the laboratory to the moment the verified test results are released and made available to the ordering clinician [2].

Depending on where the cycle starts, such as test ordering, phlebotomy, or laboratory receipt, TAT can have a different definition. There are three types of requests: surgical TAT (turnaround time for STAT surgical cases), urgent TAT, and routine TAT, as well as preanalytical (preparation for analysis), analytical, and postanalytical phases of the procedure (reporting to action) [2, 4]. Our laboratory claims responsibility for all processes of sample processing, from phlebotomy through reporting.

Weak core laboratory performance, which causes delays in diagnosis and treatment, is a barrier to providing the best possible care to patients. A study showed that there was a 43% treatment delay and a 61% increase in length of stay in the emergency department [9]. Moreover, a slow TAT can lead to an increase in requests, which results in duplication of the test [10]. This further increases the workload in the laboratory and may again increase the cost burden of the healthcare [2]. Therefore, faster TAT is universally desirable for efficient and effective management of patients in addition to saving time and money [8].

Assessing and optimizing TAT is crucial for laboratory quality management. According to ISO criteria, the laboratory must specify TATs for each of its examinations in collaboration with users to match clinical expectations [7]. The laboratory manager must regularly examine whether TAT is being maintained. When it comes to gathering objective data, the laboratory is crucial. Laboratory data makes up 60 to 70% of the objective information on a patient's record in real life [7, 10, 11].

According to various studies, laboratories are recommended to establish and regularly monitor their own turnaround time (TAT) as a critical quality indicator. However, in Ethiopia, there is limited evidence that TAT is consistently implemented and evaluated in medical laboratories. Therefore, this study was designed to assess the magnitude and associated factors of delayed TAT for hematology laboratory samples in hospitals across the South Gondar Zone, North West Ethiopia.

## 2 Methods and Materials

### 2.1 Study Design, Area and Period

**FIGURE 1:**
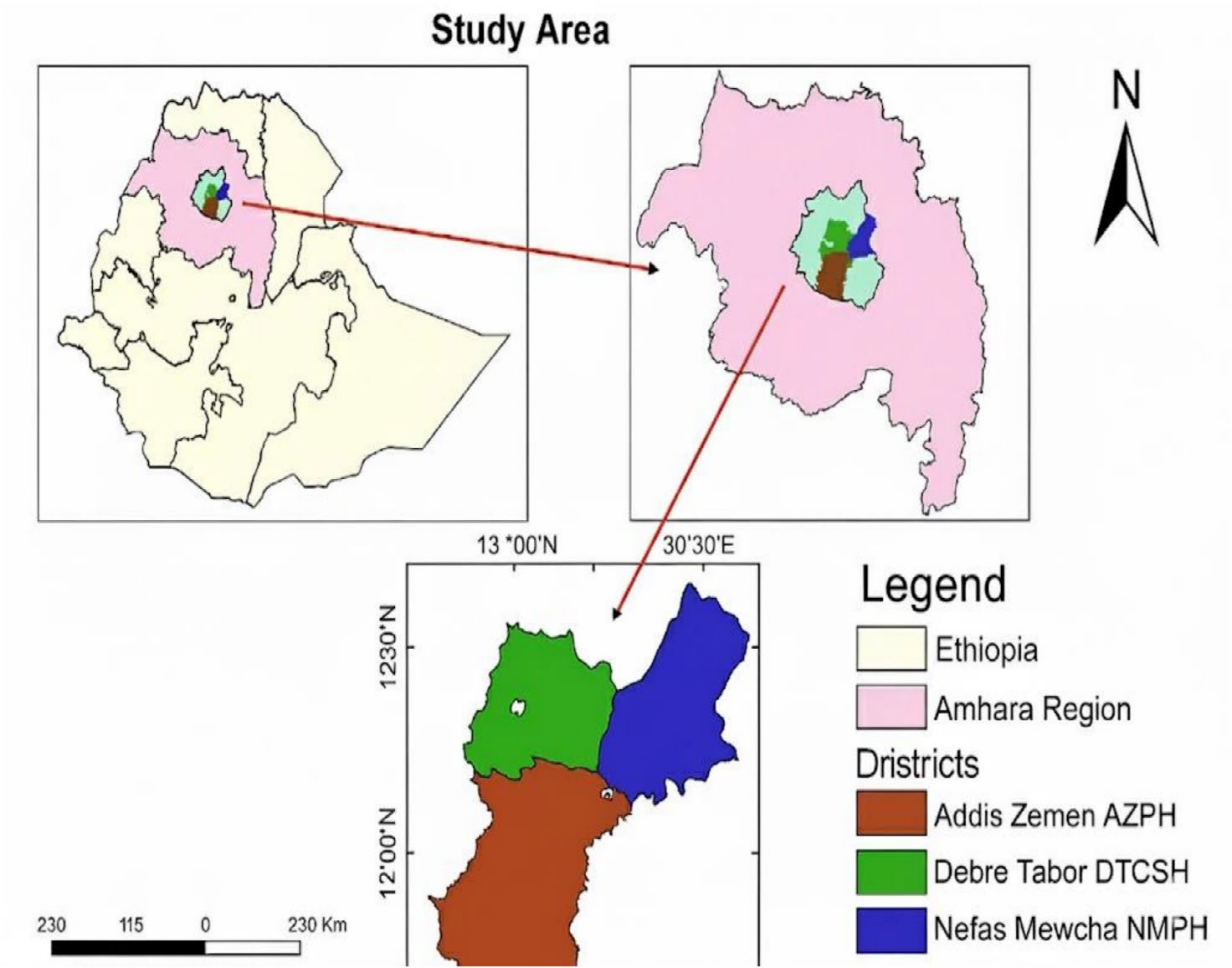
Study Area of Prolonged turnaround time of the hematology laboratory and related factors in the South Gondar Zone, North West Ethiopia, multi‐centered study.

An institutional‐based cross‐sectional study was conducted from September to December 2023 in North West Ethiopia in the medical hematology laboratory of selected South Gondar Zone hospitals. The South Gondar Zone is located in North‐West Ethiopia, in the Amhara Regional State (Figure 1). Debre Tabor Regiopolitant City is the capital city of the zone. It has a total area of 94,725 m^2^ and provides over 30 services.

According to the population census report from 2007 E C, the overall population of the South Gondar zone is around 2,578,906, with 1,041,061 males and 1,010,677 women. With a surface area of 14,095 square kilometers. There is currently one specialized referral hospital and eight primary hospitals in the South Gondar zone.

### 2.2 Study Participants

All samples TAT performed at the working hour in the hematology laboratory of the three selected hospitals during the study period were included in the study. On the other hand, samples TAT performed at the weekend, lunch break time, and night shift were excluded.

### 2.3 Sampling Method

The sample size was determined using the single population proportion formula.

*n* = Zα/2^2^ p*(1‐p)/d^2^.

We assumed a 95% confidence level, a 2% margin of error, and a 50% proportion of samples with TAT. The final determined sample size was 2401.

In the South Gondar Zone, there are nine hospitals (one comprehensive specialized referral and eight primary hospitals). Debre Tabor's specialized referral hospital was selected purposively as it is the sole referral hospital in the South Gondar Zone and provides tertiary healthcare according to Ethiopia's health tiers. From the eight primary hospitals, which are a homogeneous group providing a comparable level of healthcare, Addis Zemen and Nefas Mewcha Primary Hospitals were then selected using a simple random sampling method. Based on the flow of samples to the hematology lab (using the formula ni = [n/N] Ni), the sample size was allocated proportionally to the three hospitals: 1078 for Debre Tabor specialized referral hospital, 603 for Nefas Mewcha, and 720 for Addis Zemen hospital.

Since ni is the sample size for each hospital, n is the total sample size from three hospitals, which was 2401, N is the flow of samples to hematology in the three hospitals, which was taken from the last three months registration record, and Ni is the expected number of samples that came to the hematology laboratory of each hospital, which was predicted from the last three months; Ni for DCSRH was 3234, Ni for Addis Zemen Hospital was 2160, Ni for Nefas Mewucha Hospital was 1809, and N, which is the total samples that came to the hematology lab, was 7203.

During the study period, a systematic random sampling technique was used to pick study units from samples that came to the hematology laboratory. The Kth interval (K = 3) was estimated using a list of samples that came to the hematology laboratory. The lottery approach was used to determine the first random start, and every Kth interval from that point was chosen until the final sample was completed.

### 2.4 Data Collection and Sample Processing

The TAT for each test set by the laboratories is as follows (Table 1): CBC test: 35 min, ESR test: 60 min, hemoglobin test: 5 min, blood group and Rh (Rhesus factor) test: 15 min, peripheral morphology: 50 min, C reactive protein test: 35 min [10].

**TABLE 1:** Benchmarking of hematology laboratory turnaround times (TAT).

Source	Reported median TAT or % prolonged TAT for hematological samples	Reference Standard/Cutoff	Notes/Remarks
South Gondar Zone (Local Standards)	CBC‐ 35 min, ESR‐ 60 min/, Hb – 5 min, BG/Rh‐ 15 min, Morphology‐ 50 min, CRP‐ 35 min.	Internal facility benchmark	Internal benchmarks set and used by Debre Tabor Comprehensive Specialized Hospital and other primary hospitals in the South Gondar Zone
Ethiopian National Standards [12]	Median TAT:75 min^a^	CLSI ≤ 60 Minutes	Median TAT represents a general benchmark for routine hematological samples, consistent with the scope of international guidelines listed. TAT slightly above standard: delays linked to sample transportation and workload.
Kenya (Nairobi based hospital) [13]	Median TAT: 68 min^a^	CLSI ≤ 60 Minutes	Median TAT represents a general benchmark for routine hematological samples, consistent with the scope of international guidelines listed. Minor deviation from standards; better logistics and laboratory information system reduced delay.
Uganda National Standards [14]	Median TAT: 85 min	CLSI ≤ 60 Minutes	Delays due to manual data entry and insufficient automation
Tanzania (Muhimbili Hospital) [15]	Median TAT:95 min	CLSI ≤ 60 Minutes	Workload and staffing constrains ere major causes
International Council for Standardization in Hematology^b^ [16]	≤ 60 min for routine CBC and related hematological samples	CLSI ≤ 60 Minutes	International benchmark for basic hematology labs.
WHO Recommendation^b^ [17]	≤ 90 min for CBC and related hematological samples	CLSI ≤ 60 Minutes	Allows more flexibility in low‐resource settings.

*Note:* Test TATs for hematological samples that were performed in the hematology laboratory of the three hospitals during the sample collection period were obtained from the laboratories for each test.

^a^ For the entries ‘Ethiopian National Standards’ and ‘Kenya,’ the reported median TATs represent general benchmarks for a range of routine hematological samples, aligning with the scope of other international guidelines listed in this table.

^b^ ICSH and WHO Recommendation: These international benchmarks for routine hematological samples are included to provide a broader context and are compared against the consistent CLSI reference standard of ≤ 60 minutes.

For each test request of 2401 samples, the time starting from patient sample collection (for outpatients) or the sample for the test received by the laboratory staff (for inpatient clients' samples collected by other clinicians other than the laboratory staff) to result generation was recorded.

Each test TAT performance was compared against the time set by the laboratory for that specific test. Any test performance that takes more than the time set by the laboratory is considered a prolonged TAT.

### 2.5 Operational Definition

Turnaround Time (TAT) – Prolonged/Not Prolonged:

TAT is considered prolonged when the time taken to complete a laboratory test exceeds the standard time established by the laboratory. Conversely, TAT is considered not prolonged when the test is completed within the defined standard timeframe.

## 3 Day of the Week

This variable represents the specific day on which each test was performed, used to assess daily variations in TAT.

### 3.1 Sample Collection Time (Yes/No)

This variable indicates whether the exact time of sample collection was documented on the test request form. A response of ‘Yes’ denotes that the collection time was recorded for the individual sample, whereas ‘No’ indicates that no such documentation was available at the time of observation.

### 3.2 Sample Collector Name (Yes/No)

This variable refers to the presence or absence of the name of the individual who collected the sample on the test request form. A ‘Yes’ indicates that the name was recorded, whereas a ‘No’ signifies it was not.

### 3.3 Clotted Blood Sample (Yes/No)

This variable denotes the physical status of the blood sample. A ‘Yes’ indicates that the sample was clotted and therefore unsuitable for analysis, whereas a ‘No’ indicates that the sample was not clotted.

### 3.4 Blood Fill Level of the Test Tube

This variable assesses the volume of blood in the test tube. A sample is considered under‐filled if the volume is below the half mark of the test tube, and over‐filled if it exceeds the 3/4 level. The standard fill level is typically around 3/4 of the tube's capacity [18, 19].

### 3.5 Data Quality Assurance and Management

To ensure the quality of the data, the data collection tool was assessed by professionals for appropriateness and overall evaluation. A pretest was performed on 138 samples at Bahirdar University Tibebe Gion Specialized Hospital from September 20 to September 28, 2023. After the questionnaire was filled out, it was cheeked by the researcher; some modifications to the questioner were made for unclear and difficult questions. This pretest data was not included in the analysis of this study. Training was given for four laboratory technician data collectors by the principal investigator. The completeness of the questionnaire and checklist were rechecked at the end of each day by the principal investigator. To assure the quality of the data, the questionnaire was first tested by colleagues to avoid any ambiguity in the questions. Daily supervision was conducted during the data collection period.

### 3.6 Data Processing and Statistical Analysis

Missed values (around 70) were managed through the deletion method because data is considered as missing completely at random (MCAR), and the dataset is 2331.

Data was entered into Epidata software (Version 3.0.4), cleaned, and exported into IBM Corp.'s Statistical Package for Social Science version 20 software (IBM Corp., Armonk, NY, USA) for analysis. To summarize the data, descriptive statistics such as frequencies and percentages were used. The Kolmogorov – Smirnov test and histogram were used to ensure whether the age variable was normally distributed. The data was presented using tables.

Binary logistic regression, such as bi‐variable and multivariable logistic regression analysis, was performed. The strength of the association between predictors and outcome was calculated using the crude odds ratio (COR) and adjusted odds ratio (AOR) with a 95% CI. In the bi‐variable logistic regression analysis, variables having a *p*‐value of less than 0.25 were fitted into the multivariable logistic regression analysis. Hosmer and Lemeshow's goodness of fit statistics were used to test the model's fitness. In all cases, a *P*‐value of less than 0.05 was considered statistically significant.

### 3.7 Ethical Consideration

Ethical clearance was obtained from the institutional review board (IRB) of the Institute of Health, Jimma University (Ref. No. IHRPGH/449). After ethical approval was received, permission to conduct the study was obtained from the head of the school of medical laboratory science and the chief clinical director of the three hospitals. A support letter from the Jimma University Health Science Research Coordinating Office was written to DTCSH. During data collection, only the code of the sample was used, and the privacy and identity of the research data were confidential. Patient confidentiality, equity of services, and the interests of patients were ensured during the study period. All data collected during the study was treated with strict confidentiality and used only for this study.

## 4 Result

**FIGURE 2:**
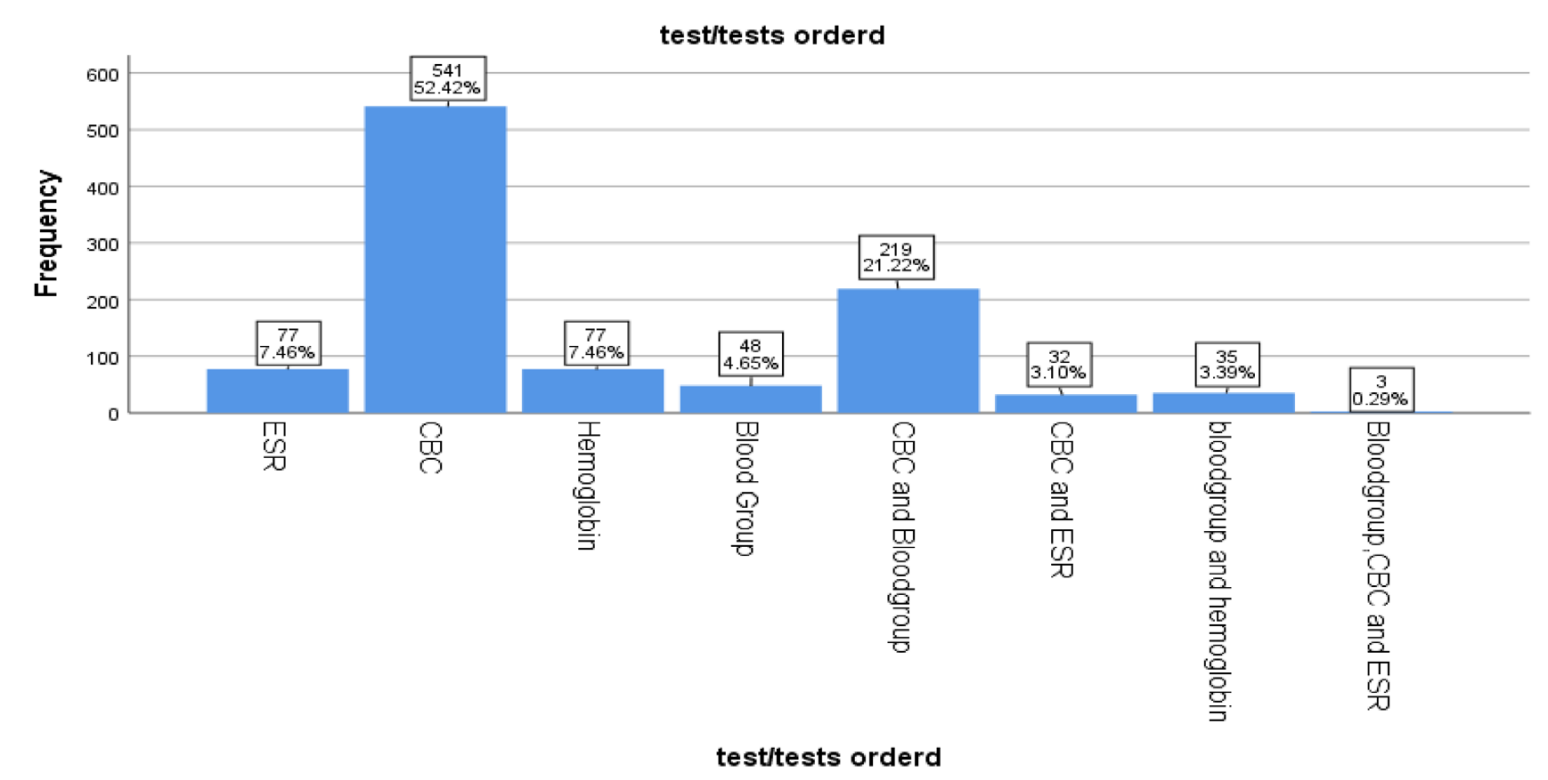
Frequency chart of test requests in hematology laboratory of DTCSH, North West Ethiopia, from September to November, 2023 (*n* = 1032).

Debre Tabor comprehensive specialized hospital: in total of 2331 hematology test requests received by the three hospitals between September and November 2023, 1032 were submitted to the hematology laboratory of Debre Tabor Comprehensive Specialized Hospital (DTCSH). Of these, Complete Blood Count (CBC) samples accounted for 541 (52.4%) of the requests. The second most commonly requested test was a combination of CBC and blood group, accounting for 219 (21.2%) (Figure 2).

**FIGURE 3:**
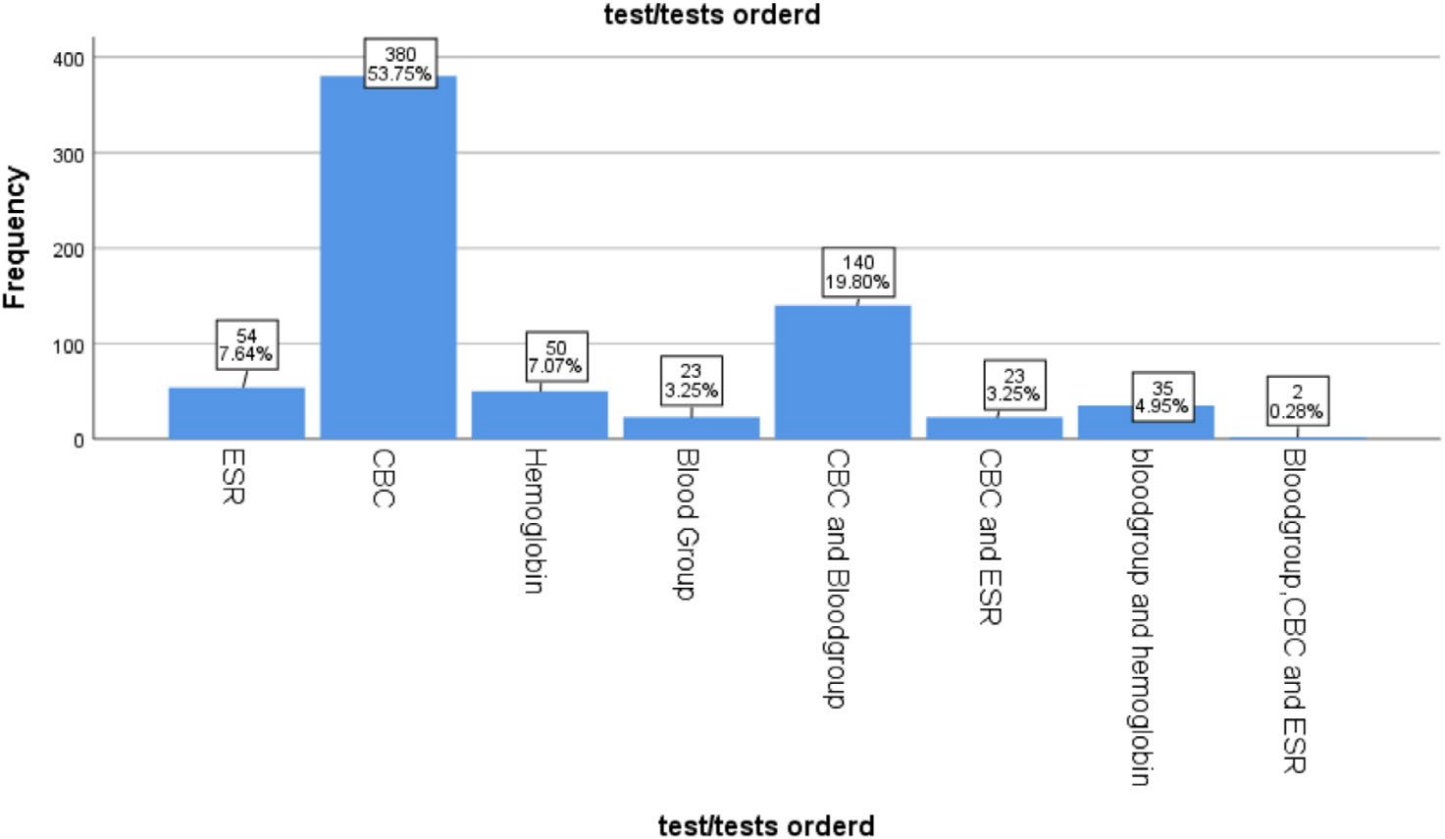
Frequency chart of test requests in hematology laboratory of AZPH, North West Ethiopia, from September to November, 2023 (*n* = 708).

Addis Zemen primary hospital: in total of 2331 hematology test requests received by the three hospitals between September and November 2023, 707 were submitted to the hematology laboratory of Addis Zemen Primary Hospital (AZPH). Of these, Complete Blood Count (CBC) samples accounted for 380 (53.7%) of the requests. The second most commonly requested test was a combination of CBC and blood group, accounting for 140 (19.8%) (Figure 3).

**FIGURE 4:**
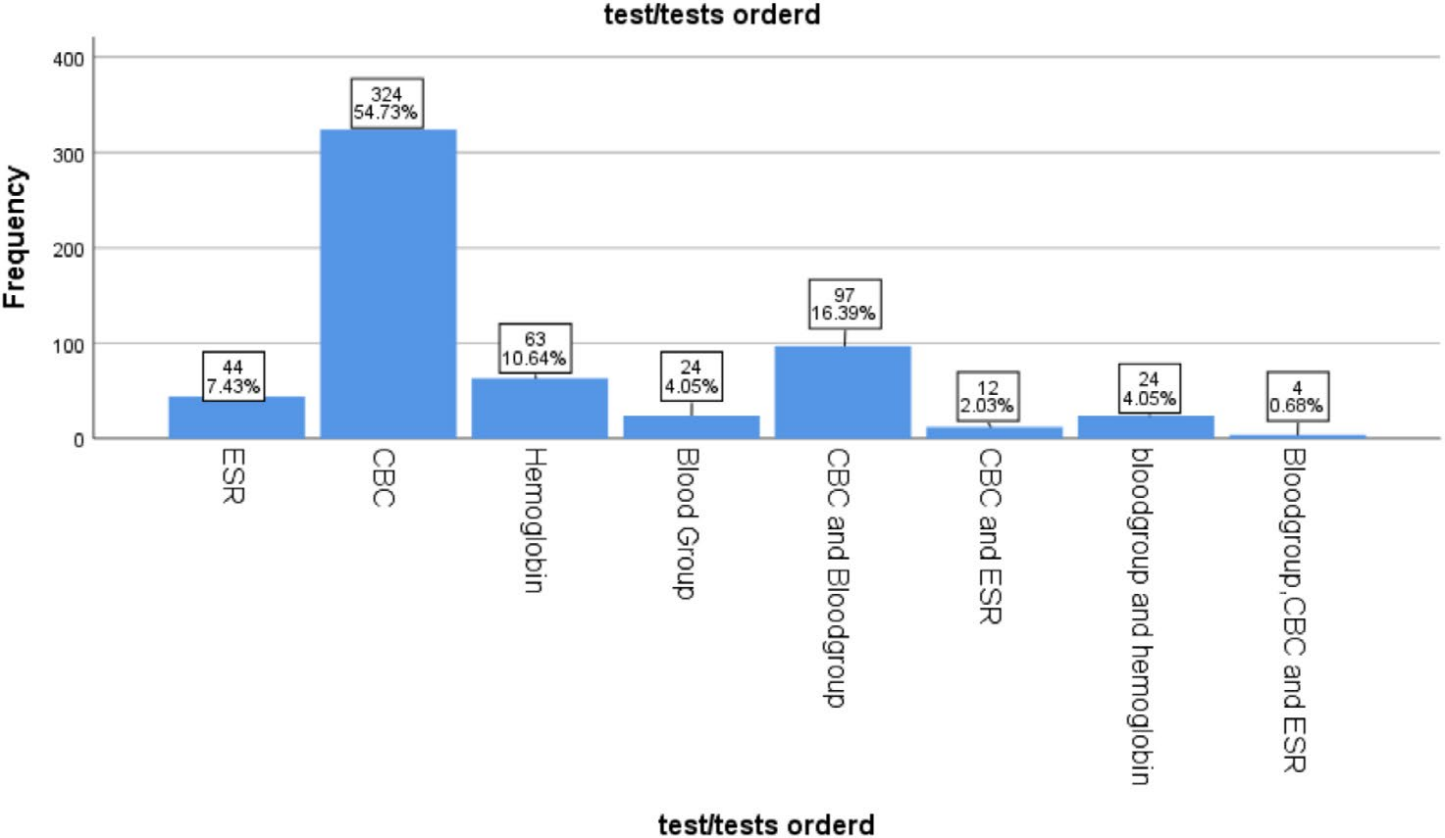
Frequency chart of test requests in hematology laboratory of NMPH, North West Ethiopia, from September to November, 2023 (*n* = 1032).

Nefas Mewcha primary hospital: in total of 2331 hematology test requests received by the three hospitals between September and November 2023, 592 were submitted to the hematology laboratory of Nefas Mewcha Primary Hospital (NMPH). Of these, Complete Blood Count (CBC) samples accounted for 324 (54.7%) of the requests. The second most commonly requested test was a combination of CBC and blood group, accounting for 97 (16.4%) (Figure 4).

**FIGURE 5:**
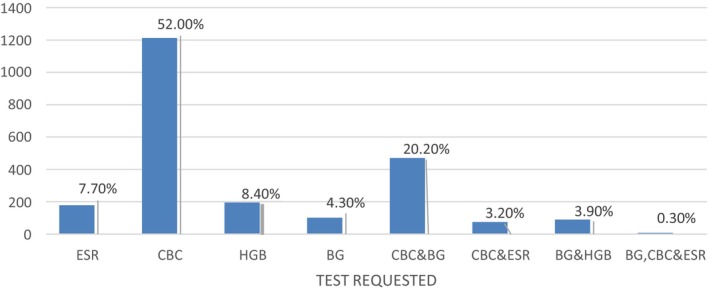
Frequency chart of test requests in hematology laboratory, North West Ethiopia, from September to November, 2023 (*n* = 2331). BG, Blood Group; CBC, Complete blood cell count, ESR, Erythrocyte sedimentation rate; HGB, hemoglobin. Samples turnaround time and related factors.

Overall: of the 2331 total test requests to the hematology laboratory from September to November, 2023, CBC/Complete Blood Count accounts for 1213 (52.0%) of the total test requests. The second most requested test was the CBC and blood group samples, with a total of 471 (20.2%) (Figure 5).

In total 2331 hematology test requests, 1032 were processed at Debre Tabor Comprehensive Specialized Hospital (DTCSH). Among these, 612 samples (59.3%) were completed within the laboratory's standard turnaround time (TAT) and thus were not considered delayed. However, 420 samples (40.7%) exceeded the standard TAT, indicating a prolonged processing time (Figure 5).

At Addis Zemen Primary Hospital (AZPH), 707 of the 2331 samples were performed. Of these, 521 samples (73.7%) were completed within the standard TAT, whereas 186 samples (26.3%) were delayed beyond the laboratory's established TAT.

**FIGURE 6:**
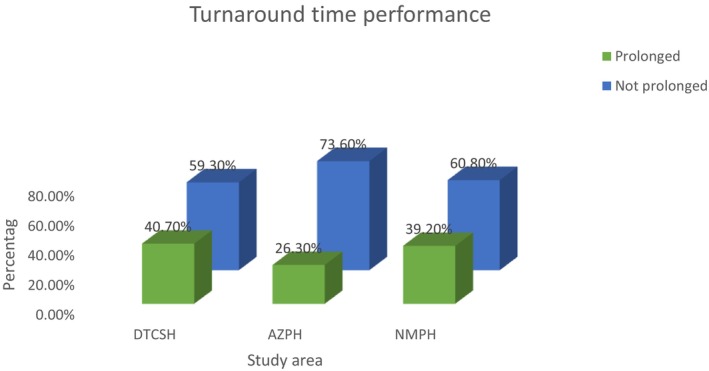
Frequency of prolonged TATs in the hematology laboratories, North West Ethiopia, From September to November, 2023 (*n* = 2331). *DTCSH—Debre Tabor comprehensive Specialized hospital, AZPH‐ Addis Zemen Primary Hospital, NMPH‐ Nefas Mewcha Primary Hospital.

At Nefas Mewcha Primary Hospital (NMPH), 592 samples were performed. Of these, 360 samples (60.8%) met the standard TAT, whereas 232 samples (39.2%) experienced prolonged turnaround times (Figure 6).

Overall, 807 of the 2331 samples (34.6%; 95% CI: 32.8%–36.6%) exceeded the standard TAT across the three hospitals (Table 2). samples.

**TABLE 2:** Frequency of prolonged TAT in the Hematology laboratories, North West Ethiopia, From September to November, 2023 (*n* = 2331).

Variables	Outcome	Count	Percentage
TAT	Prolonged	807	34.6
	Not prolonged	1524	65.4
	Total	2331	100.0

### 4.1 Sample and Related Factors That Contribute to Prolonged TAT

In total 2331 samples, 17 were delayed because of hemolysis; 5 Samples TAT was delayed due to insufficient samples; 81 Samples TAT was delayed because of clotted samples, and 243 Samples TAT was delayed because the staff was unable to process the samples on time. 320 (13.7%) of the Samples TAT was prolonged due to a machine breakdown (Table 3).

**TABLE 3:** Factors that contribute to prolonged TAT in the hematology laboratory, North West Ethiopia, From September to November, 2023 (*n* = 2331).

Variable		Count	Percentage	Cumulative percentage
Sample	Hemolysis	17	0.7	0.7
related factors	sample			
that cause	Insufficient	5	0.2	0.9
Prolonged	sample			
TAT	volume			
	not received	11	0.5	1.4
	sample			
	Unlabeled	14	0.6	2.0
	sample			
	Mislabeled	11	0.5	2.5
	sample			
	Clotted	81	3.5	6.0
	sample			
	staffs unable	243	10.4	16.4
	to process on			
	Time			
	not applicable	1940	83.6	100.0
Prolonged TAT due to machine breakdown	Yes	320	13.7	13.7
	No	2011	86.3	100

## 5 Association Factors for Prolonged TAT

To determine the association between the independent and the outcome variable, bi‐variable and multivariable binary logistic regression were done. Based on the bi‐variable logistic analysis.

Day of the Week the likelihood of prolonged TAT was significantly associated with the day of the week. Compared to Monday, samples collected on Tuesday (AOR = 0.68, 95% CI: 0.53–0.88, *p* = 0.00), Wednesday (AOR = 0.67, 95% CI: 0.52–0.87, *p* = 0.00), and Thursday (AOR = 0.76, 95% CI: 0.58–0.99, *p* = 0.049) had lower odds of experiencing prolonged TAT. Conversely, samples collected on Friday had significantly higher odds of prolonged TAT (AOR = 1.56, 95% CI: 1.21–2.01, *p* = 0.00).

Clotted Blood Samples: the presence of clotted blood was significantly associated with prolonged TAT in both unadjusted and adjusted models (AOR = 1.26, 95% CI: 1.05–1.51, *p* = 0.01), indicating that clotted samples increase the odds of delayed processing.

Blood Fill Level of the Test Tube: overfilled test tubes were significantly associated with prolonged TAT (AOR = 2.10, 95% CI: 1.25–3.55, *p* = 0.05), whereas under filled tubes showed no significant association (AOR = 1.11, 95% CI: 0.87–1.41, *p* = 0.38) (Table 4).

**TABLE 4:** Factors associated with Prolonged TAT of hematology laboratory, North West Ethiopia, from September to November, 2023 (*n* = 2331).

Variable	Category	Total samples	Prolonged TAT		COR (95% CI)	*p*	AOR (95% CI)	*p*
			Yes	No				
Day of the week	Monday (Ref)	804	297 (36.8%)	507 (33.3%)	1		1	
	Tuesday	439	123 (15.2%)	316 (20.7%)	0.66 (0.52–0.86)	0.00	0.68 (0.53–0.88)	0.00
	Wednesday	402	112 (13.9%)	290 (19.0%)	0.66 (0.51–0.86)	0.00	0.67 (0.52–0.87)	0.00
	Thursday	339	106 (13.1%)	233 (15.3%)	0.78 (0.59–1.02)	0.05	0.76 (0.58–0.99)	0.049
	Friday	347	169 (20.9%)	178 (11.7%)	1.62 (1.25–2.09)	0.00	1.56 (1.21–2.01)	0.00*
Sample	Yes (Ref)	108 (13.4%)		309 (20.3%)	1		1	
Collection								
Time	No	699 (86.6%)		1215 (79.7%)	1.65 (1.29–2.08)	0.00	1.33 (0.82–2.17)	0.25
Sample	Yes (Ref)	105 (19.4%)		296 (19.4%)	1		1	
Collector								
Name	No	702 (87.0%)		1228 (80.6%)	1.61 (1.27–2.05)	0.00	1.15 (0.69–1.88)	0.59
Clotted	No (Ref)	259 (32.1%)		570 (37.4%)	1		1	
Blood								
Sample	Yes	548 (67.9%)		954 (62.6%)	1.19 (1.002–1.143)	0.01	1.26 (1.05–1.51)	0.01*
Blood fill level of the test Tube	Appropriately filled (Ref)	121 (15.0%)		265 (17.4%)	1		1	
	Under filled	652 (80.8%)		1223 (80.2%)	1.16 (0.92–1.48)	0.19	1.11 (0.87–1.41)	0.38
	Over filled	34 (4.2%)		36 (2.4%)	2.07 (1.23–3.46)	0.00	2.10 (1.25–3.55)	0.05*

Abbreviations: AOR, Adjusted Odds Ratio; COR, Crude Odds Ratio.

* This indicates a statistical significant association where *P* < 0.05.

## 6 Discussion

Turnaround time is one of the quality indicators for quality laboratory service, prolonged turnaround time is a typical shortcoming in clinical laboratory routine practice that could jeopardize patient safety, reliability and dependability of the laboratory service, and overall quality of the clinical care practice. The goal of this study was to determine the magnitude of prolonged turnaround time and associated factors in the hematology laboratory of selected South Gondar, Ethiopia.

Appropriate and timely clinical decisions are contingent on timely reporting, which has an impact on the patient's outcome. As a result, a quick laboratory turnaround time is critical for timely patient management. As a result, analyzing this time period aids in determining the source of the delay, which is followed by a reduction in turnaround time [20].

In this study from the observed overall samples of hematology laboratory from September to November, 34.6% of test's TAT were prolonged. This finding was higher compared with the study done in University of Gondar Hospital 8.6% [21]. The possible reason for the discrepancy might be due difference in sample size, different in laboratory performance and operational workflows, and electrical fluctuation. Another reason might be sample rejection rate due to clotting was higher in our study. Staffs unable to process on time 10.4% and clotted blood sample 3.5% were the contributing factors noted in descriptive logs of prolonged TAT. Prolonged TAT due to machine breakdown were 13.7% the contributing factors noted in descriptive logs of prolonged TAT.

Subgroup analysis demonstrated notable variation in the proportion of prolonged TAT across the three study hospitals. The highest delay was observed at Debre Tabor Comprehensive Specialized Hospital (40.7%), followed by Nefas Mewcha Primary Hospital (39.2%), while Addis Zemen Primary Hospital had the lowest proportion (26.3%). These differences may reflect underlying disparities in infrastructure, staffing levels, workload, or resource availability among the facilities.

In Logistic regression, the present study identified several factors associated with prolonged turnaround time in the hematology laboratories testing.

Notably the day of the week had a significant influence, with Friday samples demonstrating higher odds of prolonged TAT. This may reflect increased workload accumulation, reduced staffing, or system inefficiencies as the week concludes. In contrast, Tuesday to Thursday showed significantly lower odds of delay, possibly reflecting more stable workflow during mid –week.

The presence of clotted blood samples was significantly associated with prolonged TAT. Clotting often necessitates sample rejection and recollection, which introduces procedural delays. This finding reinforces the importance of proper sample handling and timely delivery.

The blood fill level was also a relevant factor. Overfilled tubes doubled the odds of prolonged TAT, likely due to testing instrument limitations or the need for additional processing. The unexpectedly high odds of prolonged TAT with overfilled tubes can be attributed to the manual preanalytical steps required in the study laboratory setting. Overfilled tubes necessitate an additional step of manually aliquoting the blood into another tube to ensure proper sample volume for analysis, which adds to the overall processing time and introduces a potential source of error.

Interestingly, under filled tubes, though common, were not significantly associated with delays, potentially due to automated fagging systems allowing partial analysis.

Factors such as sample collection time and documentation of the collector's name did not reach statistical significance. While they may reflect procedural quality, they appear not to independently affect TAT when other variables are accounted for.

Overall, these findings emphasize the importance of proper preanalytical practices and resource allocation throughout the week to reduce diagnostic delays.

## 7 Limitation of the Study

Despite the strengths of using a prospective cross‐sectional design—which improved data completeness and reduced recall bias—this study has several limitations. First, the cross‐sectional nature limits the ability to infer causal relationships between the identified factors and prolonged turnaround time (TAT); the observed associations should be interpreted with caution. Second, potential selection bias may exist due to the inclusion of only three public hospitals, which may limit the generalizability of the findings to other settings, especially private or rural laboratories. If these facilities differ systematically from others in terms of staffing, infrastructure, or workflow, the magnitude of prolonged TAT reported here could either overestimate or underestimate the true burden in the broader region.

Moreover, although real‐time data collection minimized recall bias, the relatively short data collection period (three months) may not capture seasonal or temporal variability in laboratory performance. For instance, changes in staff availability, equipment maintenance schedules, or peak patient load periods could affect TAT, potentially introducing temporal bias. This may result in an underestimation or overestimation of the true prevalence and patterns of prolonged TAT over the course of a full year.

### 7.1 Generalizability

While this study provides valuable insights into the magnitude and determinants of prolonged turnaround time (TAT) in hematology laboratories, its generalizability is subject to certain limitations. The findings of this study may have limitations in their generalizability, as the research was conducted in three public hospitals within a specific zone. The results may not fully represent the broader healthcare system in Ethiopia, particularly private facilities or those in more urban or resource‐intensive settings. However, the inclusion of both a comprehensive specialized hospital and primary hospitals strengthens the relevance of our findings to similar public healthcare contexts in low‐resource regions. As such, while caution is warranted in extending these results to all laboratory contexts, the findings are likely applicable to comparable facilities operating under similar constraints.

## 8 Conclusion

This study revealed that over one‐third (34.6%) of hematology laboratory samples across selected hospitals in South Gondar experienced prolonged turnaround times (TAT), highlighting a substantial gap in timely diagnostic service delivery. Key factors associated with delayed TAT included the day of the week, clotted blood samples, and overfilled test tubes, underscoring the importance of strengthening preanalytical practices and operational workflows. Notable variations across hospitals suggest the need for facility‐specific quality improvement interventions.

While these findings provide valuable insight into the magnitude and drivers of prolonged TAT, they should be interpreted cautiously due to the cross‐sectional study design, potential selection bias, and limited data collection period. Nevertheless, the results are consistent with previous studies in similar settings and offer actionable evidence to inform laboratory quality improvement strategies. Further longitudinal or interventional studies are recommended to validate these associations and support sustainable improvements in laboratory efficiency and patient care.

## Author Contributions

Conceptualization and design of the study: B.M. Acquisition of data: B.M., A.F., T.T., S.D., M.E., M.W., A.B. Analysis and interpretation of data: B.M., Y.S., A.A., G.A.Y., M.E. Investigation: B.M., A.A., G.A., T.T., S.D., T.K., A.B., Methodology: B.M., Y.S., H.D., G.T., F.O., G.T., B.L., G.A.Y. Project administration: B.M., Y.S., T.E., L.W., G.A. Supervision: B.M., G.T., F.O., H.D. Validation: B.M., Y.S., H.D., G.T., F.O., A.B., A.A., M.W. Drafting the article: B.M., Y.S., G.A. Writing, reviewing and editing: B.M., Y.S., H.D., G.A. Final approval: B.M., G.A.Y., G.A.

## Funding

This study was supported by the author(s) received no specific funding for this work.

## Conflicts of Interest

The authors declare no conflicts of interest.

## Data Availability

The data that support the findings of this study are available from the corresponding author upon reasonable request.
